# Modelling of the Electrical Membrane Potential for Concentration Polarization Conditions

**DOI:** 10.3390/e24010138

**Published:** 2022-01-17

**Authors:** Kornelia M. Batko, Izabella Ślęzak-Prochazka, Andrzej Ślęzak, Wioletta M. Bajdur, Radomir Ščurek

**Affiliations:** 1 Department of Business Informatics, University of Economics in Katowice, 2B Bogucicka, 40287 Katowice, Poland; 2 Biotechnology Centre, Silesian University of Technology, Akademicka 2A, 44100 Gliwice, Poland; izabella.slezak-prochazka@polsl.pl; 3 Department of Health Science, Jan Dlugosz University, 13/15 Armia Krajowa Al., 42200 Częstochowa, Poland; aslezak52@gmail.com; 4 Faculty of Management, Częstochowa University of Technology, 35b Armia Krajowa Al., 42200 Częstochowa, Poland; 5 Department of Security Services, Faculty of Safety Engineering, VŠB-Technical University of Ostrava, ul. Lumirova 3, 70030 Ostrava, Czech Republic; radomir.scurek@vsb.cz

**Keywords:** membrane transport, membrane potential, concentration polarization, Kedem–Katchalsky equations, polymeric membrane, concentration Rayleigh number

## Abstract

Based on Kedem–Katchalsky formalism, the model equation of the membrane potential ($\Delta \psi_{s}$) generated in a membrane system was derived for the conditions of concentration polarization. In this system, a horizontally oriented electro-neutral biomembrane separates solutions of the same electrolytes at different concentrations. The consequence of concentration polarization is the creation, on both sides of the membrane, of concentration boundary layers. The basic equation of this model includes the unknown ratio of solution concentrations ($C_{i}/C_{e})$ at the membrane/concentration boundary layers. We present the calculation procedure ($C_{i}/C_{e})$ based on novel equations derived in the paper containing the transport parameters of the membrane ($L_{p}$, σ, and ω), solutions (ρ, ν), concentration boundary layer thicknesses ($\delta_{l}$, $\delta_{h}$), concentration Raileigh number ($R_{C}$), concentration polarization factor ($\zeta_{s}$), volume flux ($J_{v}$), mechanical pressure difference (ΔP), and ratio of known solution concentrations ($C_{h}/C_{l}$). From the resulting equation, $\Delta \psi_{s}$ was calculated for various combinations of the solution concentration ratio ($C_{h}/C_{l}$), the Rayleigh concentration number ($R_{C}$), the concentration polarization coefficient ($\zeta_{s}$), and the hydrostatic pressure difference (ΔP). Calculations were performed for a case where an aqueous NaCl solution with a fixed concentration of 1 mol m^−3^ ($C_{l}$) was on one side of the membrane and on the other side an aqueous NaCl solution with a concentration between 1 and 15 mol m^−3^ ($C_{h}$). It is shown that ($\Delta \psi_{s}$) depends on the value of one of the factors (i.e., ΔP, $C_{h}/C_{l}$, $R_{C}$ and $\zeta_{s}$) at a fixed value of the other three.

## 1. Introduction

Both biological and synthetic membranes are sensitive to changes in the physicochemical properties of the environment [1,2,3,4]. Therefore, membrane transport of its solution components can be governed by local fields (such as concentration, temperature, electric potential, or pressure fields) and global fields (such as gravitational or electromagnetic fields) [1,5]. Under real-world conditions (no external mixing of membrane-separated solutions), concentration polarization plays an important role, involving the formation of concentration boundary layers in solution regions immediately adjacent to the membrane [6,7,8,9]. In electrochemistry, the term concentration polarization is used to describe a set of phenomena accompanying the formation of concentration gradients in electrolyte solutions adjacent to a semipermeable solid/liquid interfacial surface during the flow of an electric current and the appearance of a boundary current [10,11,12,13,14].

The term concentration polarization is also used to describe the phenomena accompanying the formation of concentration boundary layers (CBLs) in both electrolyte and non-electrolyte solutions [3,4,7,8,9,15,16,17]. The effect of concentration polarization is to reduce membrane transport [7,8,9,16,17]. These layers are formed on both sides of electrically neutral selective membranes oriented horizontally, and the concentration (density) gradients in them are sensitive to the gravitational field [1,7,15,16,17]. The effect of this field is natural convection, which modifies the concentration fields in the membrane regions [7,15]. Its consequence is a partial restoration of concentration gradients across the membrane and increased membrane transport [3,16,17]. One of the effects of changing the concentration field are gravitational effects in passive osmotic and diffusive transport [1,18,19] and the gravielectric effect [16]. Theoretical modeling of the concentration polarization phenomenon is usually based on the Kedem–Katchalsky and Nernst-Planck equations [14,20,21,22,23].

The gravielectric effect in an electrochemical cell is a consequence of diffusion, concentration polarization, and the action of gravity [16]. In this study, we used a system in which two solutions with different NaCl or KCl solutions were separated by a synthetic membrane. The solutions were connected to Ag/AgCl electrodes using original bridges [16] or immersed directly into the solutions [24,25,26]. Furthermore, mathematical models of this effect were developed using the Kedem–Katchalsky equations [24,25,26]. In the first case, the dependence of the measured difference of electric potentials on the distance of electrodes from the membrane was eliminated. In the second case, the dependence was obvious. These studies showed, among other things, that the reversal of the mechanical pressure gradient with respect to the concentration (density) gradient has a significant effect on the value of the membrane potential difference [26].

The decrease in fluxes and thermodynamic forces due to the concentration polarization commonly found in nature, contributes to slowing down the source of entropy. This is an anti-entropic process.

The aim of this paper was to develop model equations of the membrane potential difference ($\Delta \psi_{s}$) for concentration polarization conditions based on Kedem–Katchalsky formalism. The basic equation of this model includes the unknown ratio of solution concentrations ($C_{i}/C_{e})$ at the membrane/concentration boundary layers. We present the calculation procedure ($C_{i}/C_{e})$ based on the novel equations derived in the paper containing the transport parameters of the membrane ($L_{p}$, σ, and ω), solutions (ρ, ν), concentration boundary layer thicknesses ($\delta_{l}$, $\delta_{h}$), concentration Raileigh number ($R_{C}$), concentration polarization factor ($\zeta_{s}$), volume flux ($J_{v}$), mechanical pressure difference (ΔP), and ratio of known solution concentrations ($C_{h}/C_{l})$. We used the obtained equation to calculate the characteristics $\Delta \psi_{s}=f(C_{h}/C_{l}),$ $\Delta \psi_{s}=f\left(\Delta P\right),$ and $\Delta \psi_{s}=f\left(R_{C}\right)$. The characteristics $\Delta \psi_{s}=f(C_{h}/C_{l})$ were calculated for different fixed values of ΔP, $\zeta_{s}$, and $R_{C}$. In contrast, the characteristics $\Delta \psi_{s}=f\left(R_{C}\right)$ were calculated for different fixed values of ΔP,$\;\zeta_{s}$, and $\left(C_{h}/C_{l}\right)$, while the characteristics $\Delta \psi_{s}=f\left(\Delta P\right)$ were calculated for different fixed values of $R_{C}$,$\;\zeta_{s},$ and $\left(C_{h}/C_{l}\right)$.

## 2. Materials and Mathematical Modeling

### 2.1. Membrane System

**Figure 1:**
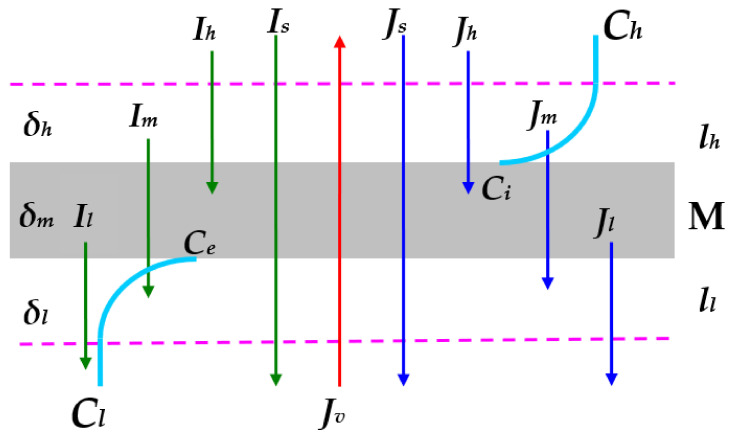
Membrane system (M—membrane; $l_{l}$ and $l_{h}$—concentration boundary layers; $C_{l}$, $C_{e}$, $C_{i}$, and $C_{h}$—solution concentrations; $J_{v}$—volume flux; *J_l_*, *J_m_*, and *J_h_*—solute fluxes; *I_l_*, *I_s_*, *I_m_*, and *I_h_*—ionic currents; *δ_l_* and *δ_h_*—concentration boundary layers thicknesses; *δ_m_*—membrane thickness.

In this paper, the considerations were based on the membrane system schematically shown in Figure 1. In this system, two mechanically unstirred solutions of the same electrolyte were separated by a hemodialyzer biomembrane (i.e., Nephrophan) made of regenerated cellulose (M), oriented in the horizontal plane. Nephrophan (Orwo VEB Filmfabrik, Wolfen, Germany) is a microporous, highly hydrophilic, isotropic, homogeneous, symmetric, and electro-neutral membrane [27]. The transport properties of a δ thick membrane are determined by the following coefficients: hydraulic conductivity ($L_{p}$), reflection (σ), solute permeability (ω), electrical conductivity (κ), and transfer number (τ). At the initial time (t=0), the concentrations of these solutions were $C_{h}$ and $C_{l}$ ($C_{h}$ > $C_{l}$). For t>0, water and solute diffusing through the membrane form on both sides of the membrane concentration boundary layers $l_{h}$ and $l_{l}$ with thicknesses of $\delta_{h}$ and $\delta_{l}$, respectively. These layers can be perceived as membranes with diffusion coefficients $D_{h}$ and $D_{l}$, reflection coefficients $\sigma_{h}$ = $\sigma_{l}$ = 0, transfer numbers $\tau_{h}$ and $\tau_{l}$, and electrical conductivity coefficients $\kappa_{h}$ and $\kappa_{l}$. The membrane together with the concentration boundary layers form the $l_{h}$/M/$l_{l}$ complex. The transport properties of this complex are determined by the following coefficients: reflection (σ), solute permeability (ω), electrical conductivity (κ), and the transfer number ($\tau_{j}$). The process of creating concentration boundary layers causes the concentration of the solution on the M/$l_{l}$ border to increase from $C_{l}$ to $C_{e}$ ($C_{e}$ > $C_{l}$) and on the $l_{h}$/M border to decrease from $C_{h}$ to $C_{i}$ ($C_{h}$ > $C_{i}$).

The solute flux and ionic current through the $l_{l}$ layer are denoted by $J_{l}$ and $I_{l}$, respectively, through the M membrane by $J_{m}$ and $I_{m}$ and through the $l_{h}$ layer by $J_{h}$ and $I_{h}$. The volume, solute, and ion current fluxes through the $l_{h}$/M/$l_{l}$ complex are denoted by $J_{v}$, $J_{s}$, and $I_{s}$, respectively. It is worth noting that it is possible to select the concentrations of the solutions in such a way that the concentration gradient and the density gradient are parallel or antiparallel to the gravity vector. Interferometric studies have shown that the formation of the layers $l_{h}$ and $l_{l}$ ends when natural convection occurs, and the membrane system reaches a steady state.

### 2.2. Equations for Fluxes and Concentration Polarization Conditions

For electrically neutral membranes, the results of membrane transport studies of electrolyte solutions can be interpreted based on Kedem–Katchalsky formalism [22,23,27]. In this formalism, the membrane is treated as a “black box”, and its properties are described by the relationship between the thermodynamic forces (which cause the permeation of solution components through the membrane) and the thermodynamic fluxes that result from these forces. For binary electrolyte solutions, the Kedem–Katchalsky equations are of the form [23,24]:

(1) $$J_{v}=L_{p}\left[\gamma \sigma RT\left(C_{h}-C_{l}\right)\;+\;\frac{P_{E}}{\kappa }I_{m}-\Delta P\right]$$

(2) $$J_{s}=\omega RT\left(C_{h}-C_{l}\right)\;+\;\bar{C}\left(1-\sigma \right)J_{v}+\frac{\tau_{j}}{z_{j}\varkappa_{j}F}I_{m}$$

(3) $$I_{m}=-P_{E}J_{v}+\frac{\tau_{j}\kappa }{z_{j}\varkappa_{j}F}\Delta \mu_{m}+\kappa E$$

(4) $$\Delta \psi_{m}=\frac{I_{m}}{\kappa }-\frac{RT}{F}\Delta \tau ln\frac{C_{h}}{C_{l}}$$

where $J_{v}$—volume flux; $J_{s}$—solute flux; $I_{m}$—ion current; $L_{p}$, σ, and $P_{E},$ and  ω—coefficients of hydraulic conductivity, reflection, electroosmotic permeability, and solute permeability, respectively; γ—van’t Hoff coefficient; *RT*—the product of the gas constant and the absolute temperature; $C_{h}$ and $C_{l}$—solution concentrations ($C_{h}$ > $C_{l}$); κ—electrical conductivity; $\tau_{j}$, $z_{j}$, and $\varkappa_{j}$—transfer number, valence, and ion number, respectively; $\bar{C}=\left(C_{h}-C_{l}\right){\left(lnC_{h}C_{l}{}^{-1}\right)}^{-1}$ $\approx \;0.5\;\left(C_{h}+C_{l}\right)$—average concentration of the solution ($\bar{C}\approx \;0.5\;\left(C_{h}+C_{l}\right)$  is only valid when the $C_{h}$ and $C_{l}$ values are not very different); $\Delta \psi_{m}$—potential difference measured with two reversible electrodes; $\Delta \tau =\tau_{a}-\tau_{c}$, $\tau_{a}$, and $\tau_{c}$—transfer numbers of anions (a) and cations (c) in the membrane, respectively; $\tau_{a}+\tau_{c}=1$.

Equations (1)–(4) are correct for sufficiently dilute and homogeneous solutions [23,24]. As already mentioned, when the solutions are not mechanically mixed, CBLs form on both sides of the membrane [4,6,15,16,17,18]. These layers can be thought of as pseudomembranes connected in series with the material membrane. The consequence of the formation of these layers is a decrease in membrane transport, which is manifested by a decrease in volume flux, solute flux, membrane potential, and ionic current [1,4,6,15,16,17].

Based on the classical [22,23] and modified [28,29] form of Equation (2) and the amperostatic condition $\left(I_{l}=I_{h}=I_{s}=0\right)$, we can write:

(5) $$J_{l}=\frac{D_{l}}{\delta_{l}}\left(C_{e}-C_{l}\right)\;+\;\frac{1}{2}\left(C_{e}+C_{l}\right)J_{v}$$

(6) $$J_{s}=\omega \zeta_{s}RT\left(C_{h}-C_{l}\right)\;+\;\frac{1}{2}\left(C_{h}+C_{l}\right)\left(1-\sigma \right)J_{v}$$

(7) $$J_{h}=\frac{D_{h}}{\delta_{h}}\left(C_{h}-C_{i}\right)\;+\;\frac{1}{2}\left(C_{h}+C_{i}\right)J_{v}$$

(8) $$J_{v}=L_{p}\Delta P-L_{p}\zeta_{s}\sigma RT\left(C_{h}-C_{l}\right)$$

where $\zeta_{s}$—the concentration polarization coefficient, $J_{vp}=L_{p}\Delta P$—the hydraulic volume flux, and $J_{vo}=L_{p}\zeta_{s}\sigma RT\left(C_{h}-C_{l}\right)$—osmotic volume flux.

In the steady state, the condition is fulfilled by:

(9) $$J_{l}=J_{h}=J_{s}$$

Equations (5)–(7) and (9) are used to calculate the solution stabilities at the boundaries M/$l_{l}$ ($C_{e}$) and $l_{h}$/M ($C_{i}$), assuming that $\sigma_{h}$ = $\sigma_{l}$ = 0:

(10) $$C_{e}=〚D_{l}C_{l}+\delta_{l}\left\{\zeta_{s}\omega RT\left(C_{h}-C_{l}\right)\;+\;\frac{1}{2}J_{v}\left[C_{h}\left(1-\zeta_{s}\sigma )-C_{l}\zeta_{s}\sigma \right)\right]\right\}〛{\left[D_{l}+\frac{1}{2}J_{v}\delta_{l}\right]}^{-1}$$

(11) $$C_{i}=〚\delta_{h}\left\{\zeta_{s}\omega RT\left(C_{h}-C_{l}\right)\;+\;\frac{1}{2}J_{v}\left[C_{l}\left(1-\zeta_{s}\sigma )-C_{h}\zeta_{s}\sigma \right)\right]\right\}-D_{h}C_{h}〛{\left[\frac{1}{2}J_{v}\delta_{h}-D_{h}\right]}^{-1}$$

For isoosmotic conditions ($J_{v}=0$, $\zeta_{s}\sigma RT\left(C_{h}-C_{l}\right)$ = ΔP), Equations (10) and (11) simplify to the form:

(12) $$C_{e}=C_{l}+\frac{\delta_{l}\left\{\zeta_{s}\omega RT\left(C_{h}-C_{l}\right)\right\}}{D_{l}}$$

(13) $$C_{i}=C_{h}-\frac{\delta_{h}\left\{\zeta_{s}\omega RT\left(C_{h}-C_{l}\right)\right\}}{D_{h}}$$

For $J_{vo}$ = 0, $J_{v}=J_{vp}=L_{p}\Delta P$ and therefore Equations (10) and (11) will take the form

(14) $$C_{e}=〚D_{l}C_{l}+\delta_{l}\left\{\zeta_{s}\omega RT\left(C_{h}-C_{l}\right)\;+\;\frac{1}{2}L_{p}\Delta P\left[C_{h}\left(1-\zeta \sigma )-C_{l}\zeta \sigma \right)\right]\right\}〛{\left[D_{l}+\frac{1}{2}L_{p}\Delta P\delta_{l}\right]}^{-1}$$

(15) $$C_{i}=〚\delta_{h}\left\{\zeta_{s}\omega RT\left(C_{h}-C_{l}\right)\;+\;\frac{1}{2}L_{p}\Delta P\left[C_{l}\left(1-\zeta \sigma )-C_{h}\zeta \sigma \right)\right]\right\}-D_{h}C_{h}〛{\left[\frac{1}{2}L_{p}\Delta P\delta_{h}-D_{h}\right]}^{-1}$$

### 2.3. Equations for the Concentration Rayleigh Number

The process of creating concentration boundary layers is controlled by the Rayleigh concentration number ($R_{C}$) [15]. For the membrane system in question, containing concentration boundary layers $l_{l}$ and $l_{h}$ with thicknesses $\delta_{l}$ and $\delta_{h}$, the expressions for the concentration Rayleigh numbers take the forms:

(16) $$R_{Cl}=g\alpha_{l}\beta_{l}{\left(\delta_{l}\right)}^{4}{\left(D_{l}\nu_{l}\right)}^{-1}$$

(17) $$R_{Ch}=g\alpha_{h}\beta_{h}{\left(\delta_{h}\right)}^{4}{\left(D_{h}\nu_{h}\right)}^{-1}$$

where *g* is acceleration due to the fact of gravity, $\rho_{l}$ and $\rho_{h}$ are the mass density of solutions ($\rho_{h}$ > $\rho_{l}$), $\nu_{l}\;$ and $\nu_{h}$ are the kinematic viscosity of the solutions ($\nu_{h}$ > $\nu_{l}$), $\delta_{l}$ and $\delta_{h}$ are the layer $l_{l}$ and $l_{h}$ thicknesses, $D_{l}$ and $D_{h}$ are the diffusion coefficients in the layers $l_{l}$ and $l_{h}$, $\nu_{l}$ and $\nu_{h}$ are the kinematic viscosity coefficients ($\nu_{h}$ > $\nu_{l}$), $\alpha_{l}=\left(\partial \rho_{l}/\partial C_{l}\right)/\rho_{l}$ and $\alpha_{h}=\left(\partial \rho_{h}/\partial C_{h}\right)/\rho_{h}$ represent the change in density of a solution due to the change in the solution concentration, and $\beta_{l}=\partial C_{l}/\partial z$ and $\beta_{h}=\partial C_{h}/\partial z$ represent the density gradient along the vertical axis. When the density of the solution above the membrane is greater than the density of the solution below the membrane, convective instabilities appear in the membrane regions when the values of $R_{Cl}$ and $R_{Ch}$ exceed their critical values. For membrane transport processes, the critical value of the $R_{C}$ = 1100.6 [30]. In the linear case, that is, when $\alpha_{l}\beta_{l}$ = $\left(\rho_{e}-\rho_{l}\right){\left(\rho_{l}\delta_{l}\right)}^{-1}$ and $\alpha_{h}\beta_{h}$ = $\left(\rho_{h}-\rho_{i}\right){\left(\rho_{h}\delta_{h}\right)}^{-1}$, Equations (16) and (17) can be written in the form [31]:

(18) $$R_{Cl}=g\left(\rho_{e}-\rho_{l}\right){\left(\delta_{l}\right)}^{3}{\left(\rho_{l}D_{l}\nu_{l}\right)}^{-1}$$

(19) $$R_{Ch}=g\left(\rho_{h}-\rho_{i}\right){\left(\delta_{h}\right)}^{3}{\left(\rho_{h}D_{h}\nu_{h}\right)}^{-1}$$

where $\rho_{e}$ and $\rho_{i}$ are the solution densities at the boundaries of M/$l_{l}$ and M/$l_{h}$ ($\rho_{i}$ > $\rho_{e}$), and $\rho_{l}$ and $\rho_{h}$ are the solution densities beyond $l_{l}$ and $l_{h}$ ($\rho_{h}$ > $\rho_{l}$).

In order to estimate $\rho_{e}-\rho_{l}$ and $\rho_{h}-\rho_{i}$ at steady state, we carried out the following considerations. Assuming that for small concentrations of solutions the density–concentration dependence is linear, we can write:

(20) $$\rho =\rho_{o}+\frac{\partial \rho }{\partial C}\cdot C$$

where ρ and $\rho_{o}$ are the densities of the solution and solvent, respectively, and (∂ρ/∂C) = const. For binary solutions, the above equation takes the form:

(21) $$\rho_{e}-\rho_{l}=\frac{\partial \rho }{\partial C}\left(C_{e}-C_{l}\right)$$

(22) $$\rho_{h}-\rho_{i}=\frac{\partial \rho }{\partial C}\left(C_{h}-C_{i}\right)$$

Considering Equations (10) and (11) in Equations (21) and (22) and then in Equations (18) and (19), we obtain concentration Rayleigh numbers ($R_{Cl}$, $R_{Ch}$) for isothermal processes of passive membrane transport

(23) $$R_{Cl}=g\frac{\partial \rho }{\partial C}\delta_{l}{}^{4}\left\{\zeta_{s}\omega C_{l}RT\left(\frac{C_{h}}{C_{l}}-1\right)+\frac{1}{2}J_{v}\left[C_{h}\left(1-\zeta_{s}\sigma \right)\;-\;C_{l}\left(1+\zeta_{s}\sigma \right)\right]\right\}{\left[\rho_{l}D_{l}\nu_{l}\left(D_{l}+\frac{1}{2}J_{v}\delta_{l}\right)\right]}^{-1}$$

(24) $$R_{Ch}=g\frac{\partial \rho }{\partial C}\delta_{h}{}^{4}\left\{\zeta_{s}\omega C_{h}RT\left(1-\frac{C_{l}}{C_{h}}\right)-\frac{1}{2}J_{v}\left[C_{h}\left(1+\zeta_{s}\sigma \right)\;-\;C_{l}\left(1-\zeta_{s}\sigma \right)\right]\right\}{\left[\rho_{h}D_{h}\nu_{h}\left(D_{h}-\frac{1}{2}J_{v}\delta_{h}\right)\right]}^{-1}$$

For isoosmotic conditions ($J_{v}=0$, $\zeta_{s}\sigma RT\left(C_{h}-C_{l}\right)$ = ΔP), Equations (23) and (24) simplify to the form [15]:

(25) $$R_{Cl}=gRT\omega C_{l}\zeta_{s}\left(\frac{\partial \rho }{\partial C}\right)\left(\frac{C_{h}}{C_{l}}-1\right)\delta_{l}{}^{4}{\left(D_{l}{}^{2}\nu_{l}\rho_{l}\right)}^{-1}$$

(26) $$R_{Ch}=gRT\omega C_{h}\zeta_{s}\left(\frac{\partial \rho }{\partial C}\right)\left(1-\frac{C_{l}}{C_{h}}\right)\delta_{h}{}^{4}{\left(D_{h}{}^{2}\nu_{h}\rho_{h}\right)}^{-1}$$

For $J_{vo}$ = 0 and $J_{v}=J_{vp}=L_{p}\Delta P$; therefore, Equations (23) and (24) take the form:

(27) $$R_{Cl}=g\frac{\partial \rho }{\partial C}\delta_{l}{}^{4}\left\{\zeta_{s}\omega C_{l}RT\left(\frac{C_{h}}{C_{l}}-1\right)+\frac{1}{2}L_{p}\Delta P\left[C_{h}\left(1-\zeta_{s}\sigma \right)\;-\;C_{l}\left(1+\zeta_{s}\sigma \right)\right]\right\}{\left[\rho_{l}D_{l}\nu_{l}\left(D_{l}+\frac{1}{2}L_{p}\Delta P\delta_{l}\right)\right]}^{-1}$$

(28) $$R_{Ch}=g\frac{\partial \rho }{\partial C}\delta_{h}{}^{4}\left\{\zeta_{s}\omega C_{h}RT\left(1-\frac{C_{l}}{C_{h}}\right)-\frac{1}{2}L_{p}\Delta P\left[C_{h}\left(1+\zeta_{s}\sigma \right)\;-\;C_{l}\left(1-\zeta_{s}\sigma \right)\right]\right\}{\left[\rho_{h}D_{h}\nu_{h}\left(D_{h}-\frac{1}{2}L_{p}\Delta P\delta_{h}\right)\right]}^{-1}$$

### 2.4. Membrane Potential Equations for Concentration Polarization Conditions

Using the procedure presented in the previous paper [4], for the situation presented in Figure 1, Equation (4) can be written in the following forms:

(29) $$\Delta \psi_{l}=\frac{I_{l}}{\kappa_{l}}-\frac{RT}{F}\left(\tau_{la}-\tau_{lc}\right)ln\frac{C_{e}}{C_{l}}$$

(30) $$\Delta \psi_{m}=\frac{I_{m}}{\kappa }-\frac{RT}{F}\left(\tau_{a}-\tau_{c}\right)ln\frac{C_{i}}{C_{e}}$$

(31) $$\Delta \psi_{h}=\frac{I_{h}}{\kappa_{h}}-\frac{RT}{F}\left(\tau_{ha}-\tau_{hc}\right)ln\frac{C_{h}}{C_{i}}$$

In the steady state, the following conditions are fulfilled:

(32) $$\Delta \psi_{s}=\Delta \psi_{l}+\Delta \psi_{m}+\Delta \psi_{h}$$

(33) $$I_{l}=I_{m}=I_{h}=I_{s}=const$$

Based on Equations (29) and (30) and the condition $\tau_{l}$ = $\tau_{h}$ = $\tau_{0}$, we obtain:

(34) $$\Delta \psi_{s}=\frac{I_{s}}{\kappa_{s}}-\frac{RT}{F}\left(\Delta \tau_{0}ln\frac{C_{h}}{C_{l}}+\left(\Delta \tau -\Delta \tau_{0}\right)ln\frac{C_{i}}{C_{e}}\right)$$

where $\Delta \tau_{0}=\tau_{0a}-\tau_{0c}$, $\Delta \tau =\tau_{a}-\tau_{c}$, and $\kappa_{s}=\kappa_{l}\kappa \kappa_{h}{\left(\kappa \kappa_{h}+\kappa_{l}\kappa_{h}+\kappa_{l}\kappa \right)}^{-1}$.

In order to represent the $C_{i}/C_{e}$ ratio in a form suitable for calculation, we form the quotient of the left and right sides of Equations (10) and (11). After performing the appropriate algebraic transformations, we obtain:

(35) $$\frac{C_{i}}{C_{e}}=\frac{C_{h}}{C_{l}}\left(\frac{\alpha_{0}+\alpha_{1}J_{v}+\alpha_{2}J_{v}{}^{2}}{\beta_{0}+\beta_{1}J_{v}+\beta_{2}J_{v}{}^{2}}\right)$$

where

$$\alpha_{0}=D_{l}\left[D_{h}-\delta_{h}\zeta_{s}\omega RT\left(1-\frac{C_{l}}{C_{h}}\right)\right]$$

$$\alpha_{1}=\frac{1}{2}\left\{\delta_{l}\left[D_{h}-\delta_{h}\zeta_{s}\omega RT\left(1-\frac{C_{l}}{C_{h}}\right)\right]-D_{l}\delta_{h}\left[\frac{C_{l}}{C_{h}}\left(1-\zeta_{s}\sigma \right)+\zeta_{s}\sigma \right]\right\}$$

$$\alpha_{2}=\frac{1}{4}\delta_{l}\delta_{h}\left[\zeta_{s}\sigma -\frac{C_{l}}{C_{h}}\left(1-\zeta_{s}\sigma \right)\right]$$

$$\beta_{0}=D_{h}\left[D_{l}-\delta_{l}\zeta_{s}\omega RT\left(\frac{C_{h}}{C_{l}}-1\right)\right]$$

$$\beta_{1}=\frac{1}{2}\left\{D_{h}\delta_{l}\left[\frac{C_{h}}{C_{l}}\left(1-\zeta_{s}\sigma \right)+\zeta_{s}\sigma \right]\;-\;\delta_{h}\left[D_{l}+\delta_{h}\zeta_{s}\omega RT\left(\frac{C_{h}}{C_{l}}-1\right)\right]\right\}$$

$$\beta_{2}=\frac{1}{4}\delta_{l}\delta_{h}\left[\zeta_{s}\sigma \;-\;\frac{C_{h}}{C_{l}}\left(1-\zeta_{s}\sigma \right)\right]$$

For the denominator of this equation to be greater than zero, the following condition must be fulfilled: $\beta_{0}+\beta_{1}J_{v}+\beta_{2}J_{v}{}^{2}$ > 0. For $J_{v}$ = 0, Equation (35) takes the form:

(36) $$\frac{C_{i}}{C_{e}}=\frac{C_{h}}{C_{l}}\left\{\frac{D_{l}\left[D_{h}-\delta_{h}\zeta_{s}\omega RT\left(1-\frac{C_{l}}{C_{h}}\right)\right]}{D_{h}\left[D_{l}-\delta_{l}\zeta_{s}\omega RT\left(\frac{C_{h}}{C_{l}}-1\right)\right]}\right\}$$

For the denominator of this equation to be greater than zero, the following condition must be fulfilled:

$$\frac{C_{h}}{C_{l}}-\frac{D_{l}}{\delta_{l}\zeta_{s}\omega T}>1$$

If we neglect the osmotic effects ($J_{vo}=L_{p}\zeta_{s}\sigma RT\left(C_{h}-C_{l}\right)$ << $J_{v}=L_{p}\Delta P)$, we obtain from Equation (35):

(37) $$\frac{C_{i}}{C_{e}}=\frac{C_{h}}{C_{l}}\left(\frac{\alpha_{0}+\gamma_{1}\Delta P+\gamma_{2}{\left(\Delta P\right)}^{2}}{\beta_{0}+\varepsilon_{1}\Delta P+\varepsilon_{2}{\left(\Delta P\right)}^{2}}\right)$$

$$\gamma_{1}=\frac{1}{2}L_{p}\left\{\delta_{l}\left[D_{h}-\delta_{h}\zeta_{s}\omega RT\left(1-\frac{C_{l}}{C_{h}}\right)\right]\;-\;D_{l}\delta_{h}\left[\frac{C_{l}}{C_{h}}\left(1-\zeta_{s}\sigma \right)+\zeta_{s}\sigma \right]\right\}$$

$$\gamma_{2}=\frac{1}{4}L_{p}{}^{2}\delta_{l}\delta_{h}\left[\zeta_{s}\sigma -\frac{C_{l}}{C_{h}}\left(1-\zeta_{s}\sigma \right)\right]$$

$$\varepsilon_{1}=\frac{1}{2}L_{p}\left\{D_{h}\delta_{l}\left[\frac{C_{h}}{C_{l}}\left(1-\zeta_{s}\sigma \right)+\zeta_{s}\sigma \right]\;-\;\delta_{h}\left[D_{l}+\delta_{h}\zeta_{s}\omega RT\left(\frac{C_{h}}{C_{l}}-1\right)\right]\right\}$$

$$\varepsilon_{2}=\frac{1}{4}L_{p}{}^{2}\delta_{l}\delta_{h}\left[\zeta_{s}\sigma -\frac{C_{h}}{C_{l}}\left(1-\zeta_{s}\sigma \right)\right]$$

### 2.5. Equations for the Thickness of the Concentration Boundary Layers

The equations for $\delta_{l}$ and $\delta_{h}$ can be obtained by transforming Equations (27) and (28) into the forms:

(38) $$a_{1}\delta_{l}{}^{4}-a_{2}\delta_{l}-a_{3}=0$$

(39) $$b_{1}\delta_{h}{}^{4}+b_{2}\delta_{h}-b_{3}=0$$

where $a_{1}=g\frac{\partial \rho }{\partial C}\left\{\zeta_{s}\omega C_{l}RT\left(\frac{C_{h}}{C_{l}}-1\right)\;+\;\frac{1}{2}J_{v}\left[C_{h}\left(1-\zeta_{s}\sigma \right)-C_{l}\left(1+\zeta_{s}\sigma \right)\right]\right\}$, $a_{2}=\frac{1}{2}J_{v}R_{Cl}D_{l}\rho_{l}\nu_{l}$, $a_{3}=R_{Cl}D_{l}{}^{2}\rho_{l}\nu_{l}$, $b_{1}=g\frac{\partial \rho }{\partial C}\left\{\zeta_{s}\omega C_{h}RT\left(1-\frac{C_{l}}{C_{h}}\right)-\frac{1}{2}J_{v}\left[C_{h}\left(1+\zeta_{s}\sigma \right)\;-\;C_{l}\left(1-\zeta_{s}\sigma \right)\right]\right\}$, $b_{2}=\frac{1}{2}J_{v}R_{Ch}D_{h}\rho_{l}\nu_{h}$, and $b_{3}=R_{Ch}D_{h}{}^{2}\rho_{h}\nu_{h}$. If we neglect the osmotic effects ($J_{vo}$ << $J_{v}$, $L_{p}\zeta_{s}\sigma RT\left(C_{h}-C_{l}\right)$ << $L_{p}\Delta P$), considering the equation $J_{v}$ = $L_{p}\Delta P$ in Equations (38) and (39) we obtain:

(40) $$e_{1}\delta_{l}{}^{4}+e_{2}\delta_{l}-e_{3}=0$$

(41) $$f_{1}\delta_{h}{}^{4}-f_{2}\delta_{h}-f_{3}=0$$

where $e_{1}=g\frac{\partial \rho }{\partial C}\left\{\zeta_{s}\omega C_{l}RT\left(\frac{C_{h}}{C_{l}}-1\right)+\frac{1}{2}L_{p}\Delta P\left[C_{h}\left(1-\zeta_{s}\sigma \right)\;-\;C_{l}\left(1+\zeta_{s}\sigma \right)\right]\right\}$, $e_{2}=\frac{1}{2}L_{p}\Delta PR_{Cl}D_{l}\rho_{l}\nu_{l}$, $e_{3}=R_{Cl}D_{l}{}^{2}\rho_{l}\nu_{l}$, $f_{1}=g\frac{\partial \rho }{\partial C}\left\{\zeta_{s}\omega C_{h}RT\left(1-\frac{C_{l}}{C_{h}}\right)-\frac{1}{2}L_{p}\Delta P\left[C_{h}\left(1+\zeta_{s}\sigma \right)\;-\;C_{l}\left(1-\zeta_{s}\sigma \right)\right]\right\}$, $f_{2}=\frac{1}{2}L_{p}\Delta PR_{Ch}D_{h}\rho_{h}\nu_{h}$, and $f_{3}=R_{Ch}D_{h}{}^{2}\rho_{h}\nu_{h}$. To obtain the equations describing $\delta_{l}$ and $\delta_{h}$ for isoosmotic conditions ($J_{v}=0$, $\zeta_{s}\sigma RT\left(C_{h}-C_{l}\right)$ = ΔP), it is sufficient to transform Equations (25) and (26) into the forms:

(42) $$\delta_{l}={\left\{R_{Cl}\nu_{l}\rho_{l}D_{l}{}^{2}{\left[gRT\omega \zeta_{s}C_{l}\left(\frac{\partial \rho }{\partial C}\right)\left(\frac{C_{h}}{C_{l}}-1\right)\right]}^{-1}\right\}}^{\frac{1}{4}}$$

(43) $$\delta_{h}={\left\{R_{Ch}\nu_{h}\rho_{h}D_{h}{}^{2}{\left[gRT\omega \zeta_{s}C_{h}\left(\frac{\partial \rho }{\partial C}\right)\left(1-\frac{C_{l}}{C_{h}}\right)\right]}^{-1}\right\}}^{\frac{1}{4}}$$

Expressions (35), (36) and (38)–(41) constitute the equations describing the membrane potential generated in a membrane system containing an isotropic, symmetric, and electron neutral polymer membrane for the conditions of concentration polarization.

## 3. Results of Calculations and Discussion

**Figure 2:**
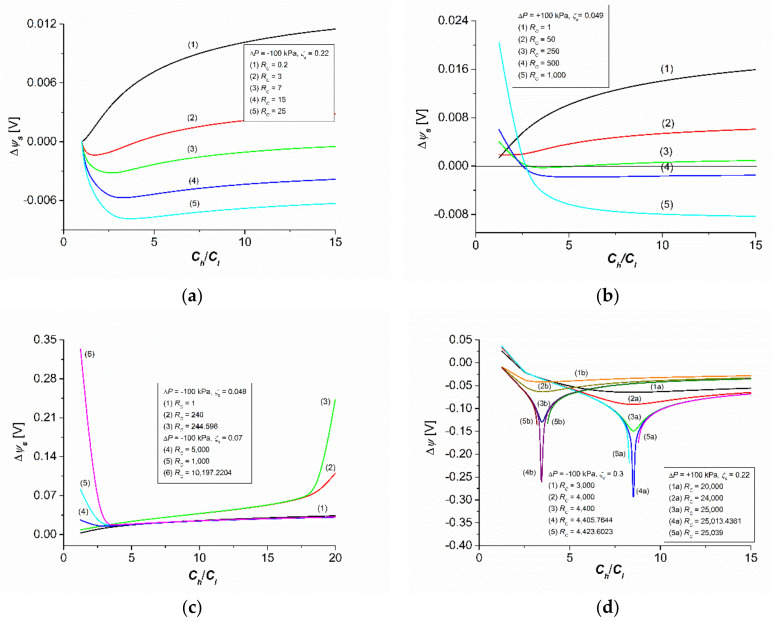
The families of the characteristics $\Delta \psi_{s}=f(C_{h}/C_{l})$ for different fixed values of ΔP,$\;\zeta_{s}$, and $R_{C}$. (**a**) characteristics of $\Delta \psi_{s}=f(C_{h}/C_{l})$ for the same ΔP = −100 kPa value and $\zeta_{s}$ = 0.22 of different set values of $R_{C}$. (**b**) characteristics of $\Delta \psi_{s}=f(C_{h}/C_{l})$ for the same ΔP = +100 kPa value and $\zeta_{s}\;$= 0.049 of different set values of $R_{C}$. (**c**) characteristics of $\Delta \psi_{s}=f(C_{h}/C_{l})$ for the same ΔP = −100 kPa value and $\zeta_{s}$ = 0.049 of different set values of $R_{C}$; characteristics of $\Delta \psi_{s}=f(C_{h}/C_{l})$ for the same ΔP = −100 kPa value and $\zeta_{s}$ = 0.07 of different set values of $R_{C}$ (**d**) characteristics of $\Delta \psi_{s}=f(C_{h}/C_{l})$ for the same ΔP = −100 kPa value and $\zeta_{s}$ = 0.3 of different set values of $R_{C}$; characteristics of $\Delta \psi_{s}=f(C_{h}/C_{l})$ for the same ΔP = +100 kPa value and $\zeta_{s}$ = 0.22 of different set values of $R_{C}$.

**Figure 3:**
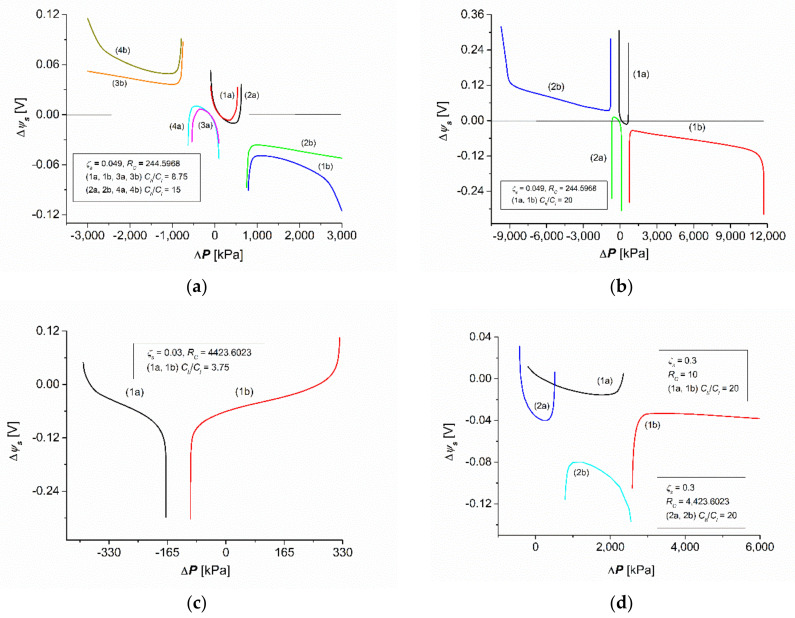
The families of the characteristics $\Delta \psi_{s}=f\left(\Delta P\right)$ for different fixed values of $C_{h}/C_{l}$, $\zeta_{s}$, and $R_{C}$. (**a**) characteristics of $\Delta \psi_{s}=f\left(\Delta P\right)$ for the same $R_{C}$ = 244.5968 value and $\zeta_{s}$ = 0.049 of different set values of $\;C_{h}/C_{l})$. (**b**) characteristics of $\Delta \psi_{s}=f\left(\Delta P\right)$ for the same $R_{C}$ = 244.5968 value and $\zeta_{s}$ = 0.049 of different set values of $\;C_{h}/C_{l}$ = 20. (**c**) characteristics of $\Delta \psi_{s}=f\left(\Delta P\right)$ for the same $R_{C}$ = 4423.6023 value and $\zeta_{s}$ = 0.03 of different set values of $\;C_{h}/C_{l}$ = 3.75. (**d**) characteristics of $\Delta \psi_{s}=f\left(\Delta P\right)$ for the same value $\zeta_{s}$ = 0.3 and $C_{h}/C_{l}$ = 20 of different set values of $\;R_{C}$.

**Figure 4:**
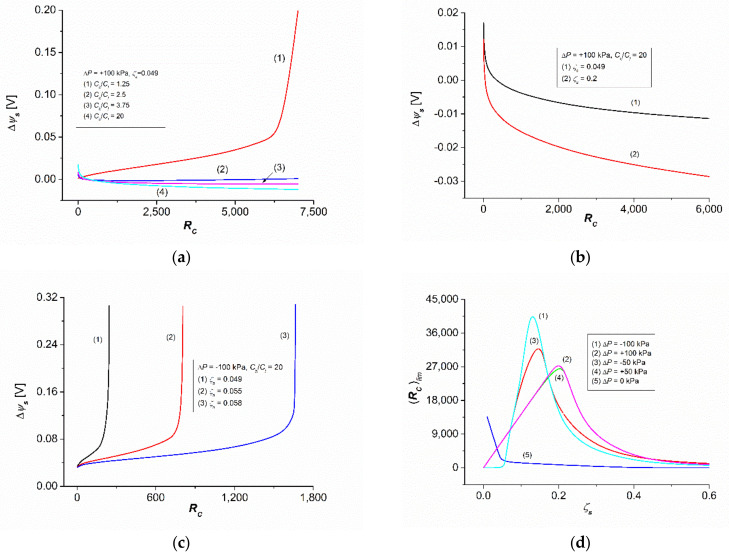
The families of the characteristics $\Delta \psi_{s}=f\left(R_{C}\right)$ for different fixed values of ΔP,$\;\zeta_{s}$ and $C_{h}/C_{l}$. (**a**) characteristics of $\Delta \psi_{s}=f\left(R_{C}\right)$ for the same ΔP = −100 kPa value and $\zeta_{s}$ = 0.049 of different set values of $C_{h}/C_{l}$. (**b**) characteristics of $\Delta \psi_{s}=f\left(R_{C}\right)$ for the same ΔP = +100 kPa value and $C_{h}/C_{l}$ = 20 of different set values of $\zeta_{s}$. (**c**) characteristics of $\Delta \psi_{s}=f\left(R_{C}\right)$ for the same ΔP = −100 kPa value and $C_{h}/C_{l}$ = 20 of different set values of $\zeta_{s}$. (**d**) characteristics of ${\left(R_{C}\right)}_{lim}=f\left(\zeta_{s}\right)$ for the different set values of ΔP.

Calculations of the membrane potential ($\Delta \psi_{s}$) were made based on Equations (34), (35), (40) and (41) for a membrane system characterized as follows. The membrane was positioned on a horizontal plane and separated by two aqueous NaCl solutions with concentrations of $C_{l}$ = 1 mol m^−3^ and $C_{h}$ = nΔC, where ΔC = 1.25 mol m^−3^ and n = 1, 2, …, 8. For the density ($\rho_{h},\;\rho_{l}$) and kinematic viscosity ($\nu_{h},\;\nu_{l}$) the following relationships were satisfied: $\rho_{h}\;$= $\rho_{l}$ + nΔρ and $\nu_{h}$ = $\nu_{l}$ + nΔν, where $\rho_{l}$ = 997.4 kg m^−3^, Δρ = 0.03.4 kg m^−3^, $\nu_{l}$ = 997.7 × 10^−9^ m^2^s^−1^, Δν = −0.03 × 10^−9^ m^2^s^−1^, and n = 1, 2, …, 8. The concentration gradient density calculated on the basis of the above data was (∂ρ/∂C) = 0.026 kg mol^−1^. It was assumed that the diffusion coefficient of NaCl in the range of tested solution concentrations was constant ($D_{l}$ = $D_{h}=D$) and, therefore, the table value D = 1.57 × 10^−9^ m^2^s^−1^ was used for the calculations. For the calculations, the transport parameters of the Nephrophan membranes used as an element of the hemodialyzer were used [32] for NaCl with the following values: $L_{p}$ = 5 × 10^−12^ m^3^N^−1^s^−1^, σ = 0.06, ω = 1.43 × 10^−9^ mol N^−1^s^−1^, Δτ = 0.39, and $\Delta \tau_{0}$ = 0.216 [16]. The values of the ζ coefficient assume values in the range $0.01\leq \zeta_{s}\leq 0.5$. Moreover, the constants R = 8.31 J mol^−1^K^−1^, g = 9.81 m s^−1^, and F = 9.65 × 10^5^ C mol^−1^ were used for the calculations. The calculations were made using the MATLAB software package. All calculations were made for isothermal conditions (T = 295 K). The characteristics $\Delta \psi_{s}=f(C_{h}/C_{l})$ for different set values of ΔP,$\;\zeta_{s}$, and $R_{C}$ are shown in Figure 2. The characteristics $\Delta \psi_{s}=f\left(R_{C}\right)$ for different set values of ΔP,$\;\zeta_{s}$, and $\left(C_{h}/C_{l}\right)$ are shown in Figure 3. Figure 4 shows the characteristics of $\Delta \psi_{s}=f\left(\Delta P\right)$ for different fixed values of $R_{C}$,$\;\zeta_{s}$, and $\left(C_{h}/C_{l}\right)$.

### 3.1. Concentration Dependence of Membrane Potential

Figure 2a shows the characteristics of $\Delta \psi_{s}=f(C_{h}/C_{l})$ for the same ΔP value and $\zeta_{s}$ of different set values of $R_{C}$. This figure shows that the curves (1)–(5) start from a point with the coordinates $C_{h}/C_{l}$ = 1 and $\Delta \psi_{s}$ = 0. Curve (1) shows that $\Delta \psi_{s}$ is positive over the entire range of the $C_{h}/C_{l}$ values. Curve (2) shows that $\Delta \psi_{s}$ fulfilled the following conditions: $C_{h}/C_{l}$ = 3.85, $\Delta \psi_{s}$ = 0; $C_{h}/C_{l}$ < 3.85, $\Delta \psi_{s}$ < 0. For curves (3)–(5), $\Delta \psi_{s}$ < 0.

Figure 2b shows that the change of the sign of ΔP and the value of $\zeta_{s}$ and the change of the set values of $R_{C}$ caused a change in the nature of the curves, illustrating the dependence $\Delta \psi_{s}=f(C_{h}/C_{l})$. This figure shows that the curves (1)–(5) started from different positive values of $\Delta \psi_{s}$. The curves (1)–(3) show that $\Delta \psi_{s}$ was positive for the entire range of $C_{h}/C_{l}$ values. The course of the curves (4) and (5) shows that $\Delta \psi_{s}$ fulfilled the following conditions: $C_{h}/C_{l}$ ≈ 2.65, $\Delta \psi_{s}$ = 0; $C_{h}/C_{l}$ < 2.65, $\Delta \psi_{s}$ < 0; $C_{h}/C_{l}$ > 2.65, $\Delta \psi_{s}$ > 0. For curves (3)–(5), $\Delta \psi_{s}$ < 0.

Figure 2c shows two families of the characteristics $\Delta \psi_{s}=f(C_{h}/C_{l})$. The first is illustrated by the curves (1)–(3), the second by the curves (4)–(6). The course of the curves (1)–(3) shows that with the increase in the value of $C_{h}/C_{l}$, the value of $\Delta \psi_{s}$ increased nonlinearly. The shape of the curves for the same $C_{h}/C_{l}$ values depended on the $R_{C}$ value. Curve (1), in the tested interval $C_{h}/C_{l}$, was a characteristic of the saturation type. The change in the type of the mentioned characteristic from (1) to (2) and (3), controlled by the $R_{C}$ value, occurred for $C_{h}/C_{l}\;$≥ 18.

The average slope of the characteristics $\Delta \psi_{s}=f{\left(C_{h}/C_{l}\right)}_{\Delta P,\;\zeta_{s},\;R_{C}}$ can be calculated from the expression $\alpha_{s}={\left[\partial \left(\Delta \psi_{s}\right)/\partial \left(C_{h}/C_{l}\right)\right]}_{\Delta P,\zeta_{s}R_{c}}$. For 1 ≤ $C_{h}/C_{l}$ < 18, the value of $\alpha_{s}$ was constant and approximately $\alpha_{s}$ = 1.65 mV (for curve (1)), $\alpha_{s}$ = 3.5 mV (for curve (4)), and $\alpha_{s}$ = 4 mV (for curve (3)). For $C_{h}/C_{l}$ ≥ 18, the value of $\alpha_{s}$ was constant and amounted to approximately $\alpha_{s}$ = 2 mV (for curve 1), $\alpha_{s}$ = 20 mV (for curve (2)), and $\alpha_{s}$ = 85 mV (for curve (3)). This means that for $R_{C}$ > 200, there was an almost abrupt increase in the value of $\alpha_{s}$. The comparison of curves (1) and 3 for $C_{h}/C_{l}$ > 18 shows that the value of the $\alpha_{s}$ coefficient for curve (3) was 42.5 times greater than the value of the $\alpha_{s}$ coefficient for curve (1). In turn, curves (4)–(6) show that that with the increase in the value of $\Delta \psi_{s}$, $C_{h}/C_{l}$ decreased nonlinearly, and for $C_{h}/C_{l}$ = 3.48, it reached the minimum value of $\Delta \psi_{s}$ = 0.225 V. For $C_{h}/C_{l}$ > 3.48, $\Delta \psi_{s}$ increased. Similarly, as in the previous case, it was possible to calculate the average slope of the characteristics $\Delta \psi_{s}=f{\left(C_{h}/C_{l}\right)}_{\Delta P,\;\zeta_{s},\;R_{C}}$. For 1 ≤ $C_{h}/C_{l}$ > 3.48, the value of $\alpha_{s}$ for curves (4)–(6) was constant and amounted to approximately $\alpha_{s}$ = 1.11 mV. For $C_{h}/C_{l}$ < 3.48, the value of $\alpha_{s}$ was constant and approximately $\alpha_{s}$ = −167 mV (for curve (3)), $\alpha_{s}$ = −44 mV (for curve 2), and $\alpha_{s}$ = −7.1 mV (for curve (1)). The comparison of curves (1) and (3) for $C_{h}/C_{l}$ < 3.48 shows that the value of the $\alpha_{s}$ coefficient for curve (3) was 23.5 times greater than the value of the $\alpha_{s}$ coefficient for curve (1).

Figure 2d shows two groups of characteristics for +ΔP (curves (1a)–(5a)) and –ΔP (curves (1b)–(5b)). These characteristics differed from the characteristics shown in Figure 2a–c because curves (1a),(2a), (3a), (4a), (1b), (2b), (3b) and (4b), illustrating the dependencies $\Delta \psi_{s}=f(C_{h}/C_{l}),$ had minima $\Delta \psi_{s}$, for which the value decreased as the $R_{C}$ value increased. The curves (5a) and (5b) did not have minima, because for $R_{C}$ = 4423.6023 (curves (5b)) and $R_{C}$ = 0.6023 (curves (5a)). This is because Equation (31) has no solutions in the set of real numbers.

### 3.2. Hydrostatic Pressure Dependencies of Membrane Potential

Figure 3 shows the characteristics $\Delta \psi_{s}=f\left(\pm \Delta P\right)$ calculated for the set values of $\zeta_{s}$ = 0.049, $R_{C}$ = 244.5968, $C_{h}/C_{l}\;$= 8.75 (curves (1a,b) and (3a,b) in Figure 3a), and $C_{h}/C_{l\;}$ = 15 (curves (2a,b) and 4a,b in Figure 3a)). These figures show that in the tested interval (i.e., $\zeta_{s}$, $R_{C}$ and $C_{h}/C_{l})$, Equation (31) had two solutions. Let us consider the curves (2a,b) shown in Figure 3a, illustrating two solutions for Equation (31) in the set of real numbers. The coordinates of the point determining the minimum of curve (1a) are as follows: $\Delta \psi_{s}$ = 0.103 V and ΔP = 0.478 MPa. In turn, the coordinates of the point determining the maximum of curve (1b) are as follows: $\Delta \psi_{s}$ = −0.064 V and ΔP = 1.275 MPa. The coordinates of the point determining the minimum of the curve (2a) are $\Delta \psi_{s}$ = 0.1 V and ΔP = 0.5 MPa. In turn, the coordinates of the point determining the maximum of curve 2b are as follows: $\Delta \psi_{s}$ = −0.086 V and ΔP = 1.55 MPa. This means that the change from $C_{h}/C_{l}$ = 8.75 to $C_{h}/C_{l}$ = 15 shifts curve (2a) in relation to curve (1a) towards higher values of Δ*P* and lower values of $\Delta \psi_{s}$. In turn, the change from $C_{h}/C_{l}$ = 8.75 to $C_{h}/C_{l}$ = 15 shifts curve (2b) in relation to curve (1b) towards lower values of ΔP and higher values of $\Delta \psi_{s}$. Moreover, curves (1a) and (2a) are symmetrical to curves (3a) and (4a) as are curves (1b) and (2b) to curves (3b) and (4b).

Curve (1a), as depicted in Figure 3b, shows that the range of changes $\Delta \psi_{s}$ falls on a relatively narrow range ΔP that meets the condition −78 ≤ ΔP ≤ 700 kPa. In the case of the curve (1b) presented in this figure, it follows that the range of changes $\Delta \psi_{s}$ is within the wide range ΔP, which satisfies the condition −780 kPa ≤ ΔP ≤ 11.7 MPa. Curve (1a) had $\Delta \psi_{s}$ had a minimum at the coordinate $\Delta \psi_{s}$ = −0.012 V and ΔP = 0.545 MPa. In turn, curve (1b) had a maximum at the coordinates $\Delta \psi_{s}$ = −0.034 V and ΔP = 1.052 MPa.

Figure 3c shows the characteristics $\Delta \psi_{s}=f\left(\Delta P\right)$ calculated for the set values $\zeta_{s}$ = 0.3, $R_{C}$ = 4423.6023, and $C_{h}/C_{l}$ = 3.75 (curves (1a,b) in Figure 3c). This figure shows that in the tested interval (i.e., $\zeta_{s}$, $R_{C}$ and $C_{h}/C_{l}$), Equation (31) had two solutions. The first is illustrated by curve (1a), and the second is shown by curve (1b). The curves presented in Figure 3c show that the curve (1a) was asymmetric in relation to curve (1b). Curve (1a) presented in Figure 3c shows that the range of changes $\Delta \psi_{s}$ was within the ΔP interval, which satisfies the condition −300 ≤ ΔP ≤ −170 kPa. In this range ΔP, the value of $\Delta \psi_{s}$ decreased nonlinearly from $\Delta \psi_{s}$ = 0.05 V to $\Delta \psi_{s}$ = −0.3 V. In turn, in the case of curve (1b), the range of changes $\Delta \psi_{s}$ was within the range ΔP, satisfying the condition −100 ≤ ΔP ≤ +300 kPa. In this case, the value of $\Delta \psi_{s}$ decreases nonlinearly from $\Delta \psi_{s}$ = −0.3 to $\Delta \psi_{s}$ = +0.107 V.

Curve 1a, as depicted in Figure 3d, shows that the range of changes $\Delta \psi_{s}$ was in the ΔP interval, which satisfies the condition −0.45 ≤ ΔP ≤ +0.514 MPa. This curve had a minimum at the point with the coordinates $\Delta \psi_{s}$ = −0.04 V and ΔP = 0.3 MPa. Moreover, this curve shows that with the increase in ΔP, the value of $\Delta \psi_{s}$ decreased nonlinearly until reaching the minimum value and then increased nonlinearly. On the other hand, in the case of curve (1b), the range of changes of $\Delta \psi_{s}$ was within the ΔP range, which satisfies the condition of 0.78 ≤ ΔP ≤ +2.54 MPa. This curve had a maximum at the coordinates $\Delta \psi_{s}$ = −0.08 V and ΔP = 1.12 MPa. Moreover, this curve shows that with the increase in ΔP, the value of $\Delta \psi_{s}$ increased nonlinearly until reaching the maximum value and then decreased nonlinearly.

The curves 2a,b shown in Figure 3d illustrate the dependence $\Delta \psi_{s}=f\left(\Delta P\right)$, calculated based on Equation (31) for the determined values $\zeta_{s}$ = 0.3, $R_{C}$ = 10, and $C_{h}/C_{l}$ = 20. This figure shows that in the examined interval $(i.e.,\;\;\zeta_{s}$, $R_{C}$ and $C_{h}/C_{l}$), Equation (31) had two solutions, which are graphically illustrated by the curves (2a,b). Curve (2a) shows that the range of changes $\Delta \psi_{s}$ was in the Δ*P* range, which satisfies the condition of −280 kPa ≤ ΔP ≤ +2.33 MPa. This curve had a minimum at the point with coordinates $\Delta \psi_{s}$ = −0.015 V and (ΔP = 1.85 MPa). Moreover, this curve shows that with the increase in ΔP, the value of $\Delta \psi_{s}$ decreased nonlinearly until reaching the minimum value and then increased nonlinearly. On the other hand, in the case of curve (1b), the range of changes of $\Delta \psi_{s}$ was within the ΔP range, which satisfies the condition of 2.6 ≤ ΔP ≤ 6.0 MPa. This curve had a maximum at the coordinates $\Delta \psi_{s}$ = −0.032 V and ΔP = 3.5 MPa. Moreover, this curve shows that with the increase in ΔP, the value of $\Delta \psi_{s}$ increased nonlinearly until reaching the maximum value and then decreased nonlinearly.

### 3.3. Rayleigh Number Dependencies of Membrane Potential

Figure 4 shows the characteristics of $\Delta \psi_{s}=f\left(R_{C}\right)\;$ for various fixed values of ΔP,$\;\zeta_{s}$, and $C_{h}/C_{l}\;$. Figure 4a shows $\Delta \psi_{s}=f\left(R_{C}\right)$ for the same value ΔP = +100 kPa and $\zeta_{s}$ = 0.049 for different set values of $C_{h}/C_{l}$. The calculation results illustrated by the curve presented in Figure 4a show that the value of $\Delta \psi_{s}$ increased nonlinearly with the increase in the $R_{C}$ value. This means that this increase was different in different $R_{C}$ ranges. For 0 < $R_{C}$ < 6333.3, the tangent of the slope of the tangent to the curve segment was approximately constant and amounted to $\Delta \left(\Delta \psi_{s}\right)$/$\Delta R_{C}$ = 6.8 μV. In turn, for 6333.3 ≤ $R_{C}$ ≤ 6729.2, the tangent of the slope of the tangent to the curve segment was approximately constant and amounted to $\Delta \left(\Delta \psi_{s}\right)$ = 0.27 mV. Hence, it follows that the ratio of the tangent slopes of the tangents to the discussed sections of curve (1), as shown in Figure 4a, was approximately 400. This means that after crossing the point with the coordinates $\Delta \psi_{s}$ = 0.04 V and $R_{C}$ = 6333.3, the kinetics of membrane transport change abruptly. It should be noted that for the tested $R_{C}$ range, all $\Delta \psi_{s}$ values were positive.

Curves (2–4), depicted in Figure 4a, show that an increase in the value of $C_{h}/C_{l}$ while maintaining the values of ΔP and $\zeta_{s}$, changes the course of the characteristic $\Delta \psi_{s}=f\left(R_{C}\right)$ from nonlinearly increasing (curve in Figure 3a) to nonlinearly decreasing (curves 2, 3, and 4 in Figure 4a). Moreover, for the tested $R_{C}$ range, the $\Delta \psi_{s}$ values were both positive and negative. It should also be noted that in the case of curve (2), in the range of small $R_{C}$ values, as the $R_{C}$ value increased, $\Delta \psi_{s}$ initially decreased quickly and then, after reaching the minimum value, ($\Delta \psi_{s}$)*_min_* = −0.002 V for $R_{C}$ = 1800, slowly grew. Curves (3) and (4) are nonlinearly decreased, but in the case of curve (4), the decrease in the value of the potential $\Delta \psi_{s}$ caused by the increase in the value of $R_{C}$ was significantly faster than in the case of curve (3).

Figure 4b shows the characteristics $\Delta \psi_{s}=f\left(R_{C}\right)$ calculated for the set values ΔP = +100 kPa, $C_{h}/C_{l}$ = 20, and $\zeta_{s}$ = 0.049 (curve (1)) and $\zeta_{s}$ = 0.049 (curve (2)). The course of these curves shows that an increase in the $R_{C}$ value causes a nonlinear decrease in the value of $\Delta \psi_{s}$. Moreover, $\Delta \psi_{s}$ takes both positive and negative values. This decrease in value is greatest for 0 < $R_{C}$ < 265 and it decreases for higher values of $R_{C}$. It should be noted that an increase in the value of $\zeta_{s}$ shifts the curves towards smaller values of $\Delta \psi_{s}$.

Figure 4c shows the characteristics $\Delta \psi_{s}=f\left(R_{C}\right)$ calculated for the set values ΔP = −100 kPa, $C_{h}/C_{l}$ = 20, and $\zeta_{s}$ = 0.049 (curve 1), $\zeta_{s}$ = 0.055 (curve (2)), and $\zeta_{s}$ = 0.058 (curve 3). The course of these curves shows that an increase in the $R_{C}$ value caused a nonlinear increase in the value of $\Delta \psi_{s}$ and that $\Delta \psi_{s}\;$ was positive. Moreover, it should be noted that an increase in the value of $\zeta_{s}$ shifted the abovementioned curves towards higher values of $R_{C}$. Within curves 1–3, three segments can be distinguished: the first—linear, the second—nonlinear, and the third—linear. The tangents to the linear segments of these curves intersected at the following coordinates: $\Delta \psi_{s}$ = 0.078 V and $R_{C}$ = 228.57 (curve (1)); $\Delta \psi_{s}$ = 0.077 V and $R_{C}$ = 779.22 (curve (2)); $\Delta \psi_{s}$ = 0.077 V and $R_{C}$ = 1641.56 (curve (3)). This means that after crossing this point, the kinetics of membrane transport changes abruptly. The tangents of the angles of inclination of the first linear sections of curves (1–3) were, respectively, Δ($\Delta \psi_{s}$)/$\Delta R_{C}$ = 0.19, 0.056, and 0.026 mV. This means that the value of this tangent decreased with the increasing value of $R_{C}$. In turn, the tangents of the angles of inclination of the second linear sections of curves (1–3) were, respectively, Δ($\Delta \psi_{s}$)/$\Delta R_{C}$ = 20, 19, and 17 mV. In addition, in this case, the value of this tangent decreased with an increasing $R_{C}$ value. These data also show that the tangent ratio of the angles of inclination for the second and first sections of the curves 1–3 were 105.339 and 654, respectively. This means that the value of this ratio decreased with an increasing value of $R_{C}$.

Figure 4d shows the characteristics ${\left(R_{C}\right)}_{lim}=f{\left(\zeta_{s}\right)}_{\Delta P}$, calculated for the different set values of ΔP. The curves in this figure are of a similar shape. The values of ($R_{C}{)}_{lim}$ illustrated by curve (1) were independent of $\zeta_{s}$ for 0 ≤ $\zeta_{s}$ ≤ 0.05. For $\zeta_{s}$ > 0.05, the value of *(*$R_{C}{)}_{lim}$ increased until the maximum value was reached at the point with coordinates ${\left(R_{C}\right)}_{lim}$ = 0.83 and $\zeta_{s}$ = 0.122. After reaching the maximum value, the value of ${\left(R_{C}\right)}_{lim}$ decreased nonlinearly until reaching ${\left(R_{C}\right)}_{lim}$ = 0, for $\zeta_{s}$ > 0.6. The values of ${\left(R_{C}\right)}_{lim}$ illustrated by curve (2) increased until the maximum value was reached at the point with coordinates ${\left(R_{C}\right)}_{lim}$ = 0.2 and $\zeta_{s}$ = 0.206. After reaching the maximum value, the value of ${\left(R_{C}\right)}_{lim}$ decreased nonlinearly until reaching ${\left(R_{C}\right)}_{lim}$ = 0, for $\zeta_{s}$ > 0.6.

In summary, the results of calculations based on Equations (34), (35), (40) and (41), presented in Figure 2, Figure 3 and Figure 4, show that the membrane potential for concentration polarization conditions depends on the value of one of the four factors—ΔP, ($C_{h}/C_{l\;}$), $R_{C}$, and $\zeta_{s}$—at fixed values of the other three.

### 3.4. Discussion

Equations (1)–(3) show that for $I_{m}$ = 0, four processes were involved in the creation of the characteristics shown in Figure 1, Figure 2, Figure 3 and Figure 4: hydraulic flow, osmosis, diffusion, and advection. In the studied range of $C_{h}$ and $C_{l}$, the role of the osmotic agent in the creation of $\Delta \psi_{s}$ was negligibly small. To demonstrate this, let us consider the equation $J_{vo}=L_{p}\zeta_{s}\sigma RT\left(C_{h}-C_{l}\right),$ describing the osmotic flux. Considering in this equation, the data $L_{p}$ = 5 × 10^−12^ m^3^N^−1^s^−1^, σ = 0.06, RT = 2.45 × 10^3^ J mol^−1^, and ΔC = 12.5 mol m^−3^, we obtain $J_{vo}$ = 0.9 × 10^−8^ m s^−1^. For isoosmotic conditions, $J_{vo}$ = $J_{vp}=L_{p}\Delta P$. This condition corresponds to $\Delta P_{p}$ = 1.8 kPa. For ΔP >> $\Delta P_{p}$ and $J_{vo}$ << $J_{vp}$. This means that the influence of osmosis on the creation of $\Delta \psi_{s}$ was negligibly small and the mechanical pressure was large. In order to show what the contribution of the diffusion and advection factor in the creation of $\Delta \psi_{s}$, let us consider Equations (1) and (2). If we consider Equation (1) in Equation (2) we obtain:

(44) $$J_{s}=J_{sd}-J_{sao}+J_{sap}=\left[\omega -L_{p}\sigma \bar{C}\left(1-\sigma \right)\right]RT\left(C_{h}-C_{l}\right)+\bar{C}\left(1-\sigma \right)L_{p}\Delta P$$

where $J_{sd}$—diffusion flux, $J_{sao}$—advective (osmotic) flux, and $J_{sap}$—advective (hydraulic) flux.

By substituting appropriate values into Equation (44), it can be shown that $J_{sd}$ >> $J_{sao}$, because $J_{sd}$ = 4 × 10^−5^ mol m^−2^s^−1^, $J_{sao}$ = 3 × 10^−9^ mol m^−2^s^−1^, and $\omega \gg L_{p}\sigma \bar{C}\left(1-\sigma \right)$. This means that the effect of osmosis on diffusion and, therefore, on the creation of $\Delta \psi_{s}$ was negligibly small. In turn, $J_{sap}=$ $\bar{C}\left(1-\sigma \right)L_{p}\Delta P$. Given the relevant data, one can show that if $J_{sap}$ = $J_{sd}$ = 4 × 10^−5^ mol m^−2^s^−1^, then $\Delta P_{ap}$ = 1.9 × 10^6^ kPa. For ΔP >> $\Delta P_{ap}$, the effect of pressure advection on diffusion and, thus, on the creation of $\Delta \psi_{s}$ was large. From the above discussion, it is clear that due to the organic range of ($C_{h}/C_{l}$) and the unlimited range of ΔP, only molecular diffusion and hydraulic advection are involved in the creation of $\Delta \psi_{s}$.

## 4. Conclusions

This paper presented an equation describing the membrane potential generated in a membrane system (containing an isotropic, symmetric, and electronically inert polymer membrane) for concentration polarization conditions. This equation for currentless conditions ($I_{s}$ = 0) has the form:

(45) $$\Delta \psi_{s}=-\frac{RT}{F}\left(\Delta \tau_{0}ln\frac{C_{h}}{C_{l}}\;+\;\left(\Delta \tau -\Delta \tau_{0}\right)ln\frac{C_{i}}{C_{e}}\right)$$

(46) $$\frac{C_{i}}{C_{e}}=\frac{C_{h}}{C_{l}}\left(\frac{\alpha_{0}+\alpha_{1}J_{v}+\alpha_{2}J_{v}{}^{2}}{\beta_{0}+\beta_{1}J_{v}+\beta_{2}J_{v}{}^{2}}\right)$$

where

$$\alpha_{0}=D_{l}\left[D_{h}-\delta_{h}\zeta \omega RT\left(1-\frac{C_{l}}{C_{h}}\right)\right]$$

$$\alpha_{1}=\frac{1}{2}\left\{\delta_{l}\left[D_{h}-\delta_{h}\zeta \omega RT\left(1-\frac{C_{l}}{C_{h}}\right)\right]-D_{l}\delta_{h}\left[\frac{C_{l}}{C_{h}}\left(1-\zeta \sigma \right)+\zeta \sigma \right]\right\}$$

$$\alpha_{2}=\frac{1}{4}\delta_{l}\delta_{h}\left[\zeta \sigma -\frac{C_{l}}{C_{h}}\left(1-\zeta \sigma \right)\right]$$

$$\beta_{0}=D_{h}\left[D_{l}-\delta_{l}\zeta \omega RT\left(\frac{C_{h}}{C_{l}}-1\right)\right]$$

$$\beta_{1}=\frac{1}{2}\left\{D_{h}\delta_{l}\left[\frac{C_{h}}{C_{l}}\left(1-\zeta \sigma \right)+\zeta \sigma \right]-\delta_{h}\left[D_{l}+\delta_{h}\zeta \omega RT\left(\frac{C_{h}}{C_{l}}-1\right)\right]\right\}$$

$$\beta_{2}=\frac{1}{4}\delta_{l}\delta_{h}\left[\zeta \sigma -\frac{C_{h}}{C_{l}}\left(1-\zeta \sigma \right)\right]$$

$$\Delta \tau_{0}=\tau_{0a}-\tau_{0c},\;\Delta \tau_{m}=\tau_{ma}-\tau_{mc}$$

$$\delta_{l}={\left\{R_{Cl}\nu_{l}\rho_{l}D_{l}{}^{2}{\left[gRT\omega \zeta C_{l}\left(\frac{\partial \rho }{\partial C}\right)\left(\frac{C_{h}}{C_{l}}-1\right)\right]}^{-1}\right\}}^{\frac{1}{4}}$$

$$\delta_{h}={\left\{R_{Ch}\nu_{h}\rho_{h}D_{h}{}^{2}{\left[gRT\omega_{m}\zeta_{s}C_{h}\left(\frac{\partial \rho }{\partial C}\right)\left(1-\frac{C_{l}}{C_{h}}\right)\right]}^{-1}\right\}}^{\frac{1}{4}}$$

This is an equation that is the sum of two logarithmic expressions. The second component is particularly complex, because it contains components with different powers, ranging from fractional to quadratic. The above equation certainly has many different solutions, depending on the parameters regarding the transport properties of the membrane and the physicochemical and hydrodynamic properties of the solutions it separates. Figure 2, Figure 3 and Figure 4 show examples of solutions in the form of the dependence: $\Delta \psi_{s}=f(C_{h}/C_{l})$ for different fixed values of ΔP,$\;\zeta_{s}$, and $R_{C}$; $\Delta \psi_{s}=f\left(R_{C}\right)$ for different fixed values of ΔP,$\;\zeta_{s}$, and $\left(C_{h}/C_{l}\right)$; $\Delta \psi_{s}=f\left(\Delta P\right)$ for different fixed values of $R_{C}$,$\;\zeta_{s}$, and $\left(C_{h}/C_{l}\right)$. Certainly, the curves presented in Figure 2, Figure 3 and Figure 4 do not exhaust all types of solutions to Equation (21).

It can be seen from the relationships in these figures that changing the values of ΔP,$\;\zeta_{s}$, $R_{C}$, and/or $\left(C_{h}/C_{l}\right)$ causes a change in the type of characteristic. This means that $\Delta \psi_{s}$ is sensitive to a factor (e.g., environmental forces), the source of which is the thermodynamic environment of the membrane. Biological membranes, which are the microscale equivalent of our skin, behave in a similar but more complex way [2]. The membrane has embedded switches that respond to physical signals from the environment, relaying information to intracellular protein pathways. Almost every environmental signal recognized by a cell has a different switch in the cell membrane. What these switches have in common is a similar design and method of operation.

Undoubtedly, synthetic membranes used as an active dressing component in the treatment of hard-to-heal wounds, such as extensive burns or venous leg ulcers, are a skin substitute [32]. In this process, the membrane serves as a barrier that creates an appropriate microenvironment between the wound and the membrane surface. The physiological environment thus created contributes to faster wound healing, which has both medical and economic dimensions.

## Data Availability

The data sets for this study are available upon request from the corresponding author.

## List of Symbols

$J_{v}$	volume flux (m s^−1^)
*J_l_*, *J_m_*, *J_h_*	solute fluxes; (mol m^−2^s^−1^)
*I_l_*, *I_s_*, *I_m_*, *I_h_*	ionic currents (A)
$L_{p}$	hydraulic conductivity coefficient (m^−3^N^−1^s^−1^)
σ	reflection coefficient
$P_{E}$	electroosmotic permeability coefficient (NA^−1^)
ω	solute permeability coefficient (mol N^−1^s^−1^)
γ	van’t Hoff coefficient
*R*	gas constant (J mol^−1^K^−1^)
*T*	absolute temperature (K)
κ	electrical conductivity (Ω^−1^m^−2^)
$\tau_{j}$	transfer number
$z_{j}$	valence
$\varkappa_{j}$	ion number
$\bar{C}$	average concentration of the solution (mol m^−3^)
$\Delta \psi_{m}$	potential difference measured with two reversible electrodes (V)
$\tau_{a}$, $\tau_{c}$	transfer number of anions (a) and cations (c) in the membrane
t	time (s)
$l_{h}$ and $l_{l}$	concentration boundary layers
*δ_l_*, *δ_h_*	thickness of the concentration boundary layers (m)
*δ_m_*	membrane thickness (m)
ΔP	mechanical pressure difference (Pa)
CBLs	concentration boundary layers
*g*	acceleration due to the fact of gravity (m s^−2^)
$D_{l}$, $D_{h}$	diffusion coefficients (m^2^s^−1^)
$\nu_{l}$, $\nu_{h}$	the kinematic viscosity coefficients (m^2^s^−1^)
$C_{e}$, $C_{i}$	solution concentrations at the boundaries of M/$l_{l}$ and M/$l_{h}$ (mol m^−3^)
$\rho_{e}$, $\rho_{i}$	solution densities at the boundaries of M/$l_{l}$ and M/$l_{h}$ (kg m^−3^)
$C_{l}$, $C_{h}$	solution concentrations beyond $l_{l}$ and $l_{h}$ (mol m^−3^)
$\rho_{l}$, $\rho_{h}$	solution densities beyond $l_{l}$ and $l_{h}$ (kg m^−3^)
